# Mercury Removal Using Sulfur-Decorated Chitosan Polymer Nanocomposites: Adsorption Performance and Mechanisms

**DOI:** 10.3390/polym17192585

**Published:** 2025-09-24

**Authors:** Mvula Confidence Goci, Anny Leudjo Taka, Lynwill Garth Martin, Vernon Sydwill Somerset, Michael John Klink

**Affiliations:** 1 Department of Natural Sciences, Faculty of Applied and Computer Sciences, Vaal University of Technology, Vanderbijlpark Campus, Vanderbijlpark 1911, South Africa; 2 Department of Chemistry and Biotechnology, Faculty of Science, University of Regina, 3737 Wascana Parkway, Regina, SK S4S 0A2, Canada; lytany04@yahoo.fr; 3 Cape Point Global Atmosphere Watch Station, South Africa Weather Service, c/o CSIR, Stellenbosch 7599, South Africa; 4 Chemistry Department, Faculty of Applied Sciences, Cape Peninsula University of Technology, Bellville 7535, South Africa

**Keywords:** biopolymer nanocomposites, sulfur functionalization, mercury removal

## Abstract

In this work, pCh-MWCNTs@Ag-TiO_2_/S and pCh-MWCNTs@Ag-TiO_2_ nanocomposites were synthesized through a combined phosphorylation and cross-linked polymerization method. The materials were thoroughly characterized using several analytical techniques, including SEM/EDS, FTIR, TGA, and BET analysis. SEM images revealed that the pCh-MWCNTs@Ag-TiO_2_/S nanocomposite displayed a smooth, flake-like morphology with spherical, dark greenish particles. EDS analysis confirmed the presence of Si, S, P, and Ag as prominent elements, with Ti, C, and O showing the most intense peaks. The TGA curves indicated significant weight loss between 250–610 °C for pCh-MWCNTs@Ag-TiO_2_ and 210–630 °C for pCh-MWCNTs@Ag-TiO_2_/S, corresponding to the decomposition of organic components. FTIR spectra validated the existence of functional groups such as hydroxyl (-OH), carboxyl (-COOH), and carbonyl (-C=O) on the surface of the nanocomposites. Following characterization, the materials were evaluated for their capacity to adsorb Hg^2+^ at parts-per-billion (ppb) concentrations in contaminated water. Batch adsorption experiments identified optimal conditions for mercury removal. For pCh-MWCNTs@Ag-TiO_2_, the best performance was observed at pH 4, with an adsorbent dose of 4.0 mg, initial mercury concentration of 16 ppb, and a contact time of 90 min. For pCh-MWCNTs@Ag-TiO_2_/S, optimal conditions were at pH 6, a dosage of 3.5 mg, the same initial concentration, and a contact time of 100 min. Each parameter was optimized to determine the most effective conditions for Hg^2+^ removal. The nanocomposites showed high efficiency, achieving more than 95% mercury removal under these conditions. Kinetic studies indicated that the adsorption process followed a pseudo-second-order model, while the equilibrium data aligned best with the Langmuir isotherm, suggesting monolayer adsorption behavior. Overall, this research highlights the effectiveness of sulfur-modified chitosan-based nanocomposites as eco-friendly and efficient adsorbents for the removal of mercury from aqueous systems, offering a promising solution for water purification and environmental protection.

## 1. Introduction

Mercury is a toxic heavy metal of global environmental concern due to its harmful effects on humans, animals, and plants [1,2]. Unlike many other heavy metals, mercury can easily vaporize into the atmosphere at relatively low temperatures. In the air, mercury primarily exists in three forms: gaseous elemental mercury (GEM), gaseous oxidized mercury (GOM), and particulate-bound mercury (PBM), with GEM being the most abundant, comprising approximately 95% to 99% of atmospheric mercury [3,4,5]. At present, various measures have been developed to address mercury contamination in both water and air, mainly including adsorption, chemical precipitation, ion exchange, and membrane filtration, etc. However, these conventional methods often face challenges such as high operational costs, secondary waste generation, and limited effectiveness in reducing mercury concentrations in wastewater to safe levels. Consequently, there is a growing demand for advanced materials that offer enhanced adsorption capacity, improved selectivity, and high reusability [6,7]. In response to these limitations, nanotechnology-based adsorption methods have emerged as a promising alternative due to their cost-effectiveness, operational simplicity, and enhanced removal efficiency [8,9]. Hence, nanotechnology is increasingly recognized as one of the most promising approaches for purifying water and removing environmental contaminants. In environmental remediation, nanotechnology involves the use of nanomaterials, referred to as nanosorbents that function as adsorbents to capture pollutants from water or air. These nanosorbents are engineered with nanostructures and possess pore sizes ranging from 1 to 100 nanometers, allowing them to effectively adsorb a wide range of pollutants, including inorganic and organic compounds, antimicrobial agents, pathogens, and microorganisms [10]. Moreover, nanotechnology involves the development of nanomaterials and their diverse applications across multiple areas of life [11]. This research aims to explore the use of highly porous and recyclable chitosan-based polymer nanocomposites for the remediation of mercury contamination in the environment. Chitosan, a natural polysaccharide derivative composed of linear β-(1,4)-linked N-acetylglucosamine units, has gained attention as a promising adsorbent for water treatment due to its abundance, biocompatibility, biodegradability, and the presence of functional groups such as amino and hydroxyl [12,13,14]. Recent developments in chitosan-based materials, particularly those incorporating nanomaterials and nanoparticles (e.g., MWCNTs, Ag, TiO_2_, and S), have greatly improved their performance by leveraging their intrinsic high porosity, low density, and three-dimensional (3D) structural properties [15,16,17]. These materials exhibit multifunctional characteristics that make them effective for adsorbing a wide range of pollutants from wastewater. Notably, they do not produce secondary waste and can be regenerated through suitable desorption techniques [6,18]. Enhancing the properties of these chitosan-based nanocomposites can expand their potential in diverse applications, particularly in water treatment and environmental cleanup. Furthermore, these nanocomposites are formulated from materials that are either non-toxic or exhibit low toxicity, making them environmentally sustainable, biocompatible, and reusable. The incorporation of sulfur nanoparticles further enhances their performance, making these chitosan-based nanocomposites highly effective and antimicrobial, and also allowing for more selective metal sorption [19,20].

## 2. Materials and Methods

All the purchased chemicals had a higher purity and were utilized without further treatment or purification. Table 1 lists the chemicals used in the study, their purity, and the suppliers they were sourced from.

### 2.1. Synthesis of pCh-MWCNTs@Ag-TiO_2_/S Biopolymer Based Nanocomposite

The synthesis of pCh-MWCNTs@Ag-TiO_2_/S was conducted following a step wise process.

#### 2.1.1. Functionalization of MWCNTs

MWCNTs was achieved by acid treatment using a mixture of 3:1 H_2_SO_4_ and HNO_3_ acid, as described in a previous study to obtain oxidized MWCNTs (Oxi-MWCNTs) [21].

#### 2.1.2. Phosphorylation of Chitosan

Chitosan was functionalized through the phosphorylation method described by Wang and Liu (2014) with some modifications [22].

#### 2.1.3. Synthesis of Sulfur (S) Nanoparticles

Sodium thiosulphate (STS) solution was prepared by dissolving 1.241 g of solid STS pentahydrate (MW: 248.18 g/mol) in 450 mL distilled water. For the synthesis of sulfur nanoparticles, 50 mL of 0.2 M HCl was added to 450 mL of the STS solution with stirring (300 rpm) at 50 °C for 48 h.

Na_2_S_2_O_3_ + 2HCl → 2NaCl + S + SO_2_ + H_2_O

### 2.2. Synthesis of Biopolymer: Cross-Linking Polymerization of Phosphorylated Chitosan with Oxi-MWCNTs Following by Decoration with Metal Nanoparticles

**Figure 1:**
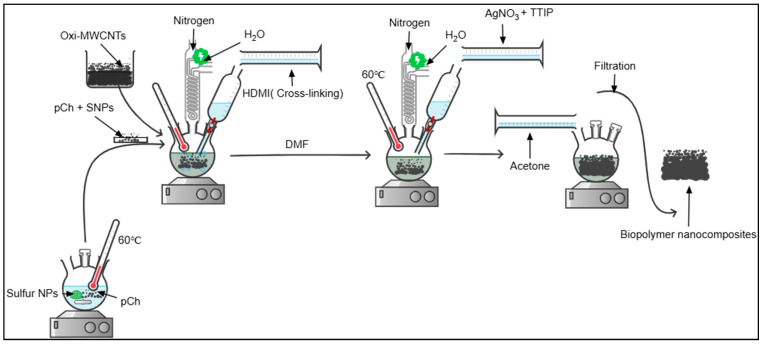
Schematic diagram of preparation of biopolymer nanocomposites.

The co-polymerization of the prepared phosphorylated chitosan with oxidized MWCNTS was done by the cross-linking polymerization method using hexamethylene diisocyanate as a cross-linking agent to obtain phosphorylated chitosan cross-linked multiwalled carbon nanotubes (pCh-MWCNTs) polymer nanocomposite as shown in Figure 1. Then, the decoration of the pCh-MWCNTs with Ag-TiO_2_ and Ag/S metal nanoparticles was achieved by the sol–gel method, as described by Taka et al., 2020 with some modifications [23].

### 2.3. Characterization Studies

#### 2.3.1. Fourier Transform Infrared Spectroscopy

FTIR spectroscopy (Perkin-Elmer, Waltham, MA, USA) was used to determine the presence of oxygen-containing groups (functional groups) on the surface of the adsorbents. The sample analysis was carried out as follows: One percent by weight of each sample to be analyzed was mixed with potassium bromide (KBr) using a mortar and pestle. Then, the mixture was pressed into a transparent pellet with a mechanical press. The resulting pellet was then placed on the sample holder of an FTIR (Perkin Elmer spectrophotometer–Spectrum 100) in which an FTIR spectrum was recorded as percent transmittance in the range 400 cm^−1^–4000 cm^−1^.

#### 2.3.2. Scanning Electron Microscope–Energy Dispersive Spectroscopy (SEM-EDS)

To determine the surface morphologies and the elemental composition of the synthesized materials, scanning electron microscopy coupled with energy dispersive spectroscopy (EDS) was employed. In these studies, samples were prepared for analysis by placing a few mg of each sample on a carbon tape. All the polymer samples were then coated with gold to make their surfaces conductive. Each sample was then mounted on the sample holder of a VEGA 3 TESCAN SEM (TESCAN, Brno, Czech Republic) which was analyzed by energy dispersive spectroscopy (EDS) with appropriate focus and magnification to determine the elemental composition of the samples.

#### 2.3.3. Thermo Gravimetric Analysis (TGA)

The types of information that can be gleaned from TGA (Perkin-Elmer Pyris Series TGA 4000 analyzer, Waltham, MA, USA) are: the thermal stability, the sample composition, the volatile components, the thermal decomposition, the effect of reactive or corrosive atmospheres, the estimated lifetime of a product, and the oxidative stability. In this work, thermal studies, sample composition, and thermal degradation were evaluated using the Pyris Series TGA 4000 analyzer. A ceramic pan containing approximately 9–10 mg of sample was placed in the instrument oven. The analysis was then performed under an oxygen gas flow of 20 mL/min and a temperature programmed from 30 °C to 900 °C at a rate of 10 °C/min.

#### 2.3.4. Dynamic Light Scattering (DLS): Surface Charge Analysis

Dynamic light scattering (DLS) is a physical technique that can be used to determine the size distribution profile of small particles in suspension or polymers in solution and was used to investigate the stability of the synthesized biopolymer nanocomposites. Using a dynamic light scatterer (Malvern Zetasizer Nanoseries, Malvern, UK) the zeta potential of the pH of the solution was measured and the zeta potential (mV) was plotted against the pH and used to determine the isoelectric point charge of the adsorbent.

#### 2.3.5. Brunauer–Emmett–Teller (BET) Surface Area Analysis

The surface area, pore volume and pore size of the synthesized polymers were determined using the Brunauer–Emmett–Teller (BET) method on a Micrometrics Tristar 3000 surface and porosity analyzer (Norcross, Norcross, GA, USA) used in nitrogen physisorption at a certain temperature and pressure. To analyze the samples, approximately 0.25 g of each sample was degassed under nitrogen at 100 °C for 4 h.

### 2.4. Hg Adsorption Studies

All stock solutions and working standards were prepared using 0.1 M HCL, to oxidize the Hg and make it soluble. A standard stock solution of Hg^2+^ (3683 × 10^−5^ M) was prepared by dissolving an appropriate amount of HgCl_2_ in a 1000 mL volumetric flask containing 0.1 M HCl. The working standard solutions (2, 4, 8, 12, 16, and 20 ppb) were prepared by appropriate dilution of the stock solutions.

Hg^2+^ adsorption studies were carried out using the pCh-MWCNTs@Ag-TiO_2_ and pCh-MWCNTs@Ag-TiO_2_/S adsorbents in batch mode. The experimental adsorption studies were carried out by adding 0.0025 g of the adsorbent to 30 mL of mercury (II) solution into a 50 mL bottle and stirring the mixture in a shaker at 150 rpm at 25 °C for 24 h. Then, the samples were collected from the shaker, filtered through 0.45 μm syringe filters (Fischer Scientific, Ottawa, ON, Canada) and the filtrates were analyzed for total gaseous mercury (TGM) concentration using a Direct Mercury Analyzer–80 (Milestone, Sorisole, Italy). All the experiments were carried out in duplicate and the average value was used for further analysis. The different factors affecting adsorption were investigated. The mercury percent removal (%*R*) and adsorption capacity ($q_{e}$) were calculated using Equations (1) and (2) [24], respectively.

(1) $$\%R=\frac{C_{i}-C_{e}}{C_{i}}\times 100$$

(2) $$q_{e}=\frac{C_{i}-C_{e}}{m}\times V$$

where *C_i_* is the initial concentration (mg/L), *C_e_* is the final concentration (mg/L), *m* is the mass of the adsorbent (g), *V* is the volume of the eluent (L), and $q_{e}$ is the amount of solute adsorbed at equilibrium (mg/g).

The effects of various parameters on the rate of the adsorption process were observed by varying pH of the solution, adsorbent dosage, contact time, adsorbent concentration, and temperature. The data obtained after batch adsorption experiments was fitted with various isotherm models (e.g., Langmuir, Freundlich) and kinetic models (e.g., pseudo-first-order and pseudo-second-order).

### 2.5. Adsorption Isotherms

The removal mechanisms and adsorption behavior of all the adsorbents in this study were ascertained using the two-parameter adsorption models, namely the Freundlich and Langmuir isotherms, which characterize the relationship between the adsorbent and the adsorbate. According to the Langmuir model, adsorption interaction between the adsorbent and adsorbate can only happen across a homogeneous surface and is limited to a monolayer coverage. The equation used in the Langmuir isotherm [24], is given as Equation (3).

(3) $$q_{e}=\frac{q_{m}bC_{e}}{\left(1+bC_{e}\right)}$$

where, *b* is the Langmuir isotherm constant (L/mg), and $q_{m}$ is the adsorption capacity at equilibrium (mg/g). The Langmuir separation factor (*R_L_*), which provides information about the type of adsorption, can be calculated using Equation (4). If the calculated *R_L_* values are between 0 and 1, the adsorption process is considered favorable, but if *R_L_* > 1, the adsorption of the adsorbate is characterized as unfavorable and if *R_L_* = 1, linear adsorption is predicted.

(4) $$R_{L}=\frac{1}{\left(1+bC_{i}\right)}$$

The Freundlich isotherm, on the other hand, shows that adsorption occurs over a heterogeneous surface with a multilayer coverage [25]. A Freundlich equation in linear form is shown as (Equation (5)) [26].

(5) $$q_{e}=k_{f}{C_{e}}^{n}$$

where *k_f_* is the Freundlich constant (L/g) and *n* is the Freundlich adsorption intensity exponent, which is the adsorption describes driving force or surface heterogeneity. The adsorption process is considered favorable when the n value is between 1 and 10 [26].

### 2.6. Kinetics Modelling

The rate-controlling steps and the adsorption mechanisms of Hg (II) by pCh-MWCNTs@Ag-TiO_2_ and pCh-MWCNTs@Ag-TiO_2_/S were investigated using pseudo-first-order (Equation (6)) and pseudo-second-order (Equation (7)) kinetic models.

(6) $$q_{t}=q_{e}({1-e}^{-k_{1}t})$$

(7) $$q_{t}=\frac{tk_{2}{q_{e}}^{2}}{\left(1+k_{2}tq_{e}\right)}$$

where $q_{t}$ (mg/g) is the adsorption capacity of the adsorbate at time (*t*), *t* is contact time (min), *k*_1_ (1/min) and *k*_2_ (g/mg·min^−1^) are rate constant for PFO and PSO, respectively.

### 2.7. Elovich Model

The Elovich model explains chemisorption processes that take place on heterogeneous surfaces in the absence of desorption, and it is widely applied to both gas–solid and liquid–solid adsorption systems [27].

(8) $$q_{t}=\frac{1}{\beta }ln\left(\alpha \beta \right)+\frac{1}{\beta }\;ln(t)$$

where $q_{t}$ is the amount of adsorbate adsorbed at time *t* (mg/g), *α* is the initial adsorption rate (mg/(g·min)), *β* is desorption constant (g/mg) related to surface coverage and activation energy, and *t* is time (min).

### 2.8. Intraparticle Diffusion

To further understand the reaction mechanisms and the rate-controlling steps of the adsorption of Hg (II) by these adsorbents, the Weber and Morris (1963) model was used [28].

(9) $$q_{t}={K_{d}t}^{0.5}+C$$

where *K_d_* (mg/(g min^0.5^)) is the intraparticle rate constant and *C* (mg/g) is the intercept that represents the boundary layer thickness. If the plot of $q_{t}$ versus *t*^0.5^ passes through the origin then intraparticle diffusion is the rate-limiting step [29].

### 2.9. Thermodynamics

The thermodynamic parameters such as enthalpy (Δ*H*°), Gibbs free energy (Δ*G*°), and entropy (Δ*S*°) were assessed to determine the spontaneity and feasibility of Hg (II) ions sorption by pCh-MWCNTs@Ag-TiO_2_ and pCh-MWCNTs@Ag-TiO_2_/S. The parameters were evaluated at different temperatures (298, 308, 318, and 328 K). The parameters were calculated from Equations (10)–(12).

(10) $$\Delta G{}^{\circ}=-RTlnK_{L}$$

where *R* is the universal gas constant (8.314 J/mol/K), *T* is the temperature (K), and *K_L_* is the partial coefficient calculated using:

(11) $$K_{L}=\frac{q_{e}\;}{C_{e}}$$

∆*H*° and ∆*S*° can be calculated using Equation (12) by plotting a van’t Hoff plot (the graph of $lnK_{L}$ against *1*/*T*).

(12) $$lnK_{L}=-\frac{∆H{}^{\circ}\;}{RT}+\frac{∆S{}^{\circ}}{R}$$

## 3. Results and Discussions

### 3.1. Characterization of the Adsorbents

#### 3.1.1. SEM-EDS

**Figure 2:**
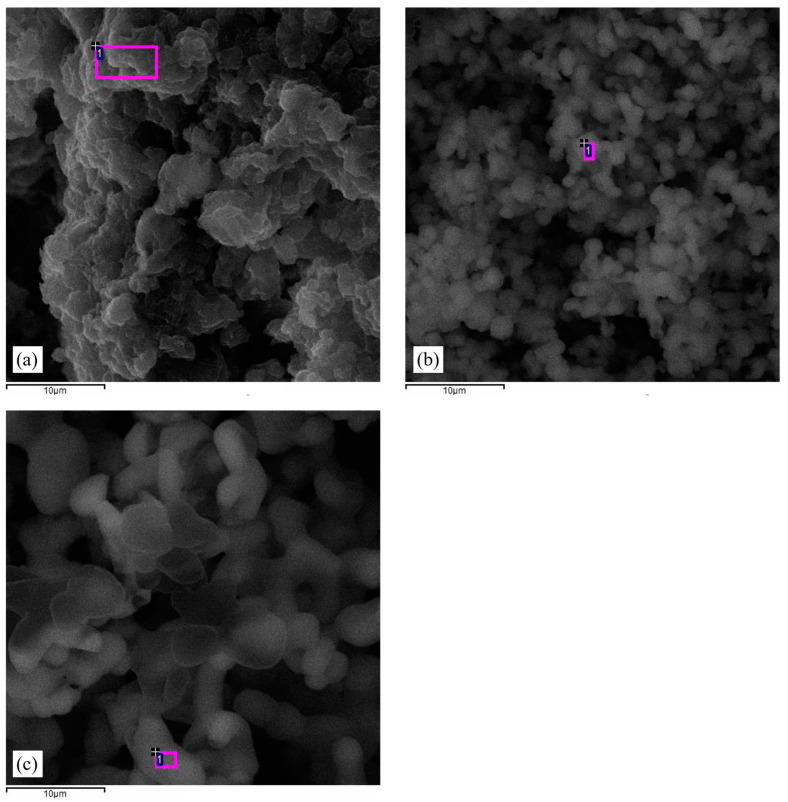
SEM micrographs of (**a**) pCh-MWCNTs@Ag-TiO_2_/S, (**b**) pCh-S, and (**c**) S.

Figure 2a–c show that the nanoparticles exhibit a spherical and agglomerated morphology, especially in the case of pCh-MWCNTs@Ag-TiO_2_/S and pCh@S. This indicates the formation of a new phase on the surface area of both nanocomposites. The SEM images of pCh-MWCNTs@Ag-TiO_2_/S (Figure 2a) show a smooth flake-like surface with a dark greenish spherical appearance, whereas those of pCh@S (Figure 2b) and SNPs (Figure 2c) were spherical but brownish and yellowish in appearance, respectively. Figure 2b shows that the surface of pCh-S consists of tiny smooth particles, while Figure 2c shows the surface of synthesized sulfur nanoparticles to be dense and yellowish.

**Figure 3:**
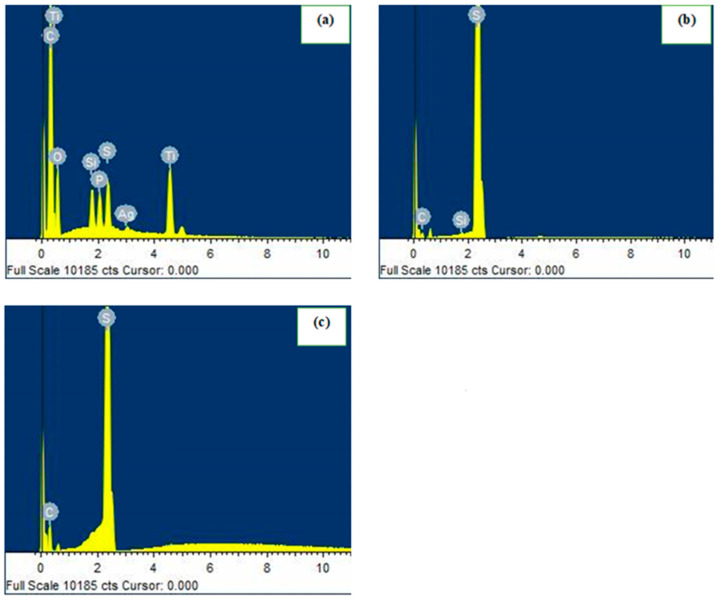
EDS spectra of (**a**) pCh-MWCNTs@Ag-TiO_2_/S, (**b**) pCh-S, and (**c**) S.

The EDS analysis is performed on selected three-point agglomerates, as shown in Figure 3a–c. The EDS spectrum of pCh-MWCNTs@Ag-TiO_2_/S (Figure 3a) show the presence of Si, S, P, and Ag as the main elements. Ti, C, and O showed the most intensive peaks, with Ti and C, being the highest peaks followed by O. In addition, 17% of the O content inferred the successful attachment of -OH groups, and 74% in C indicated that the surface of the adsorbent had polar groups such as carbonyl and carboxylic groups as shown in the FTIR spectroscopy (Figure 3a). The EDS spectra of Figure 3b,c show the presence of S, which confirmed the synthesis of pCh@S and S, and that the nanocomposite of pCh@S and S was successfully synthesized. Furthermore, the elemental composition of pCh-MWCNTs@Ag-TiO_2_/S confirms the presence of major elements by EDS analysis, and this supports that the nanocomposite of pCh-MWCNTs@Ag-TiO_2_/S was successfully prepared.

The mass of detected elements in the adsorbent pCh-MWCNTs@Ag-TiO_2_/S is listed in Table 2 and shows the percentage difference of the following elements available in the adsorbents: C (74.19%), O (17.48%), P (1.06%), Ti (4.46%), and S (1.49%). Furthermore, the presence of these elements generates charges on the surface of the as-prepared pCh-MWCNTs@Ag-TiO_2_/S and leads to electrostatic forces between the sample and Hg^2+^ in the solution.

#### 3.1.2. Fourier Transform Infrared (FT-IR) Spectroscopy

**Figure 4:**
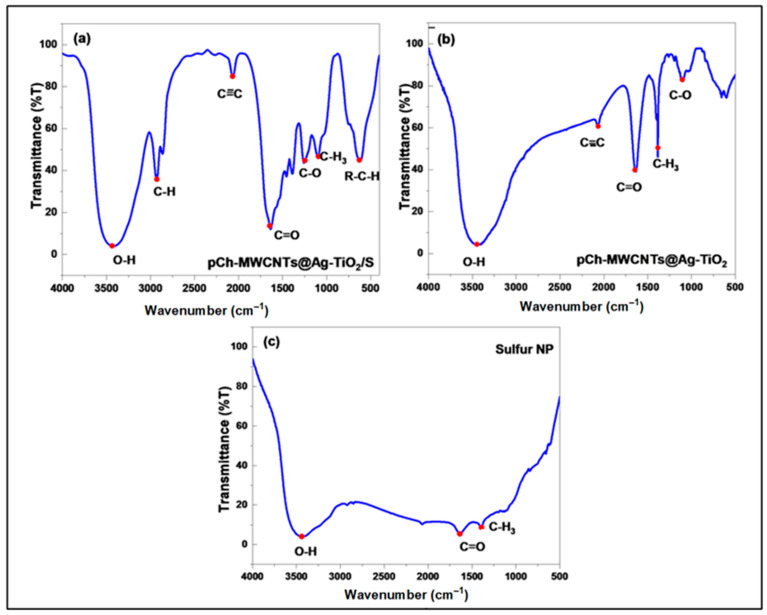
Fourier transform infrared (FTIR) spectra of (**a**) synthesized pCh-MWCNTs@Ag-TiO_2_/S, (**b**) pCh-MWCNTs@Ag-TiO_2_, and (**c**) SNPs.

Figure 4a–c show the FTIR spectra of pCh-MWCNTs@Ag-TiO_2_/S, pCh-MWCNTs@Ag-TiO_2,_ and pCh-S, respectively, in the range between 500 cm^−1^ and 4000 cm^−1^. Figure 4a shows intensive peaks, namely C-O stretching at 1254 cm^−1^–1338 cm^−1^, C-H stretching at 2927 cm^−1^, and O-H groups at 3438 cm^−1^. The presence of hydroxyl groups on the surface of pCh-MWCNTs@Ag-TiO_2_/S was due to the partial oxidation of MWCNT surfaces during the chemical modifications [30]. Furthermore, the FTIR spectrum in Figure 4a shows the presence of C=C stretch from 2057 cm^−1^ and C=O stretch from 1647 cm^−1^. These peaks indicated that the surface of the adsorbent presents polar groups, such as carbonyl and carboxylic groups. The peak found at 683 cm^−1^ is related to sulfur bonded to alkyl groups.

On the other hand, Figure 4b shows that C=O and C=C were detected for pCh-MWCNTs@Ag-TiO_2_ with intense peaks at 1638 cm^−1^ and 2064 cm^−1^, respectively. This is due to the oxidation of some carbon atoms on the surfaces of MWCNTs by HNO_3_/H_2_SO_4_ acids. The broad peak at 3441 cm^−1^ is assigned to the O-H vibration of the intermolecular and intramolecular hydrogen bonds. Among the three adsorbents, the intensity of the bands for the carboxyl and hydroxyl groups in Figure 4a is higher than those in Figure 4b,c. The results confirmed that there is a higher density of oxygen-containing functional groups on the pCh-MWCNTs@Ag-TiO_2_/S surfaces [31,32,33].

#### 3.1.3. Thermal Decomposition Studies

**Figure 5:**
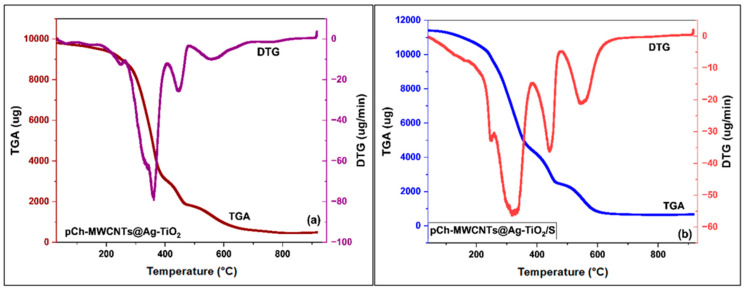
Thermogravimetric analysis (TGA) and derivative thermogravimetric analysis (DTA) of (**a**) pCh-MWCNTs@Ag-TiO_2_ and (**b**) pCh-MWCNTs@Ag-TiO_2_/S.

Thermogravimetric analysis was used to determine the thermal decomposition of pCh-MWCNTs@Ag-TiO_2_/S and pCh-MWCNTs@Ag-TiO_2_ derived from chemical activation. The weight loss profile from thermogravimetric analysis (TGA) and derivation (DTGA) is shown in Figure 5a,b. These figures show that three different parts of thermal decomposition are involved in the thermal behavior of the nanocomposites, showing the different phases of weight loss in the temperature range of 50–900 °C. The first decomposition was observed at 50–240 °C for pCh-MWCNTs@Ag-TiO_2_ (Figure 5a) and 50–210 °C for pCh-MWCNTs@Ag-TiO_2_/S (Figure 5b). This could be due to dehydration/release of moisture on the surface of the adsorbents. A continuous mass loss during 250–610 °C for pCh-MWCNTs@Ag-TiO_2_ (Figure 5a) and 210–630 °C for pCh-MWCNTs@Ag-TiO_2_/S (Figure 5b) was observed in the graph. This mass loss likely corresponds to the removal of all organic materials. This shows a difference in weight loss between the two samples. The chemically treated polymer experiences a greater weight loss because the thermal stability is affected by the modification of the polymer. This is because the stability of the polymer with sulfur improved after its modification with pCh-MWCNTs and the NPs.

#### 3.1.4. Surface Area and Pore Sizes Analysis

**Figure 6:**
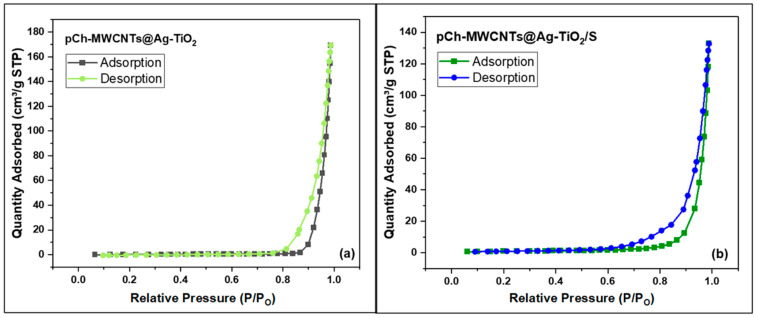
N_2_ adsorption/desorption isotherms of (**a**) pCh-MWCNTs@Ag-TiO_2_ and (**b**) pCh-MWCNTs@Ag-TiO_2_/S.

Figure 6 shows the nitrogen gas adsorption/desorption graph of both adsorbents, pCh-MWCNTs@Ag-TiO_2_ (Figure 6a) and pCh-MWCNTs@Ag-TiO_2_/S (Figure 6b). The graph shows the type of adsorption isotherm since a larger hysteresis loop associated with mesoporous structures of pCh-MWCNTs@Ag-TiO_2_ and pCh-MWCNTs@Ag-TiO_2_/S was also observed, indicating a type IV adsorption/desorption isotherm. Zhao et al. (2016) explained this phenomenon as adsorption on a mesoporous multilayer followed by capillary condensation. In addition, the P/P_o_ is <1 and shows monolayer adsorption associated with a type I adsorption/desorption isotherm [34].

Table 3 shows the results according to BET surface area, pore volume and pore size of the adsorbents. The results show that pCh-MWCNTs@Ag-TiO_2_/S has the larger surface area (3.506 m^2^/g) and pore volume (0.262 cm^3^/g). The surface area (0.578 m^2^/g) and pore volume (0.205 cm^3^/g) of pCh-MWCNTs@Ag-TiO_2_ are lower compared to pCh-MWCNTs@Ag-TiO_2_/S. This is due to the combination of the carbonaceous composition with high surface area [35]. The pore sizes of both adsorbents were above 2 nm, suggesting that the adsorbents had a mesoporous structure. In addition, Table 3 shows that the larger the surface area, the larger the pore volume, and the smaller the pore size.

#### 3.1.5. Dynamic Light Spectroscopy (DLS)

**Figure 7:**
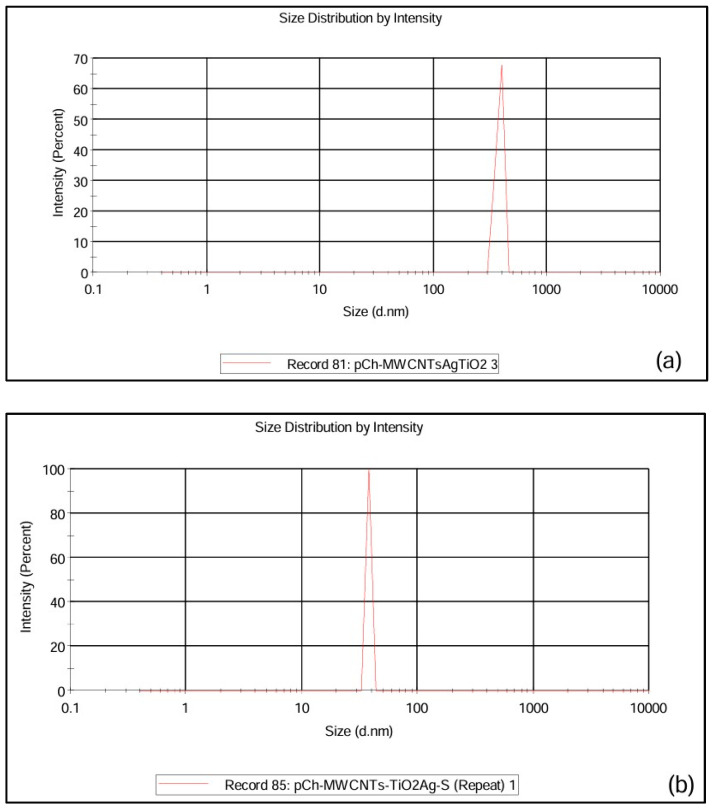
Zeta size of (**a**) pCh-MWCNTs@Ag-TiO_2_ and (**b**) pCh-MWCNTs@Ag-TiO_2_/S.

Dynamic light spectroscopy (DLS) was used to investigate the hydrodynamic particle size of the pCh-MWCNTs@Ag-TiO_2_ and pCh-MWCNTs@Ag-TiO_2_/S adsorbents and are shown in Figure 7. The average hydrodynamic diameter of pCh-MWCNTs@Ag-TiO_2_ (Figure 7a) and pCh-MWCNTs@Ag-TiO_2_/S (Figure 7b) was 2564 d.nm and 5456 d.nm, respectively. Compared to pCh-MWCNTs@Ag-TiO_2_/S, pCh-MWCNTs@Ag-TiO_2_ has a smaller average hydrodynamic diameter. The experimental results suggest or reveal that the surface modification of the adsorbent with sulfur improved the homogeneity of the polymer.

### 3.2. Adsorption Studies

#### 3.2.1. Point of Zero Charge for Adsorbents

**Figure 8:**
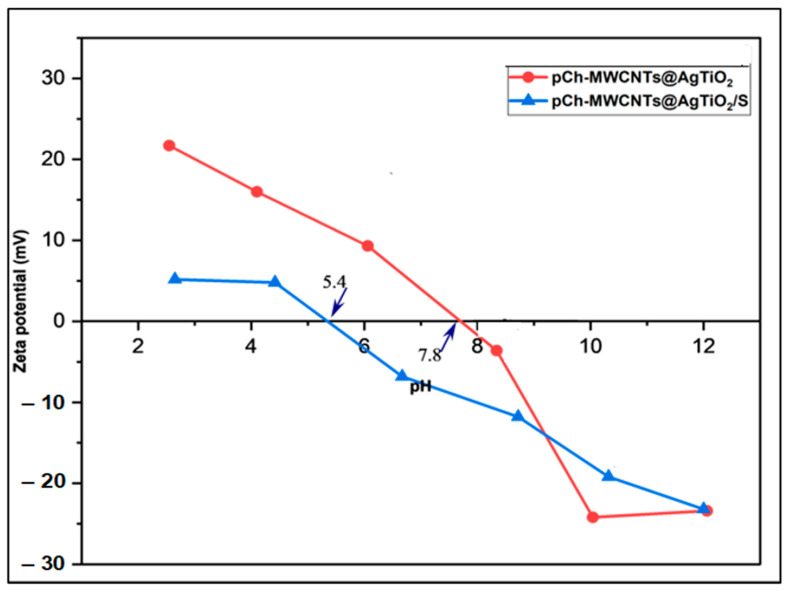
Zeta potential of pCh-MWCNTs@Ag-TiO_2_/S and pCh-MWCNTs@Ag-TiO_2_.

The zeta potential versus pH for pCh-MWCNTs@Ag-TiO_2_ and pCh-MWCNTs@Ag-TiO_2_ is depicted in Figure 8. The isoelectric point is determined from the graph as the pH point at which the surface area of the adsorbent is zero. Additionally, it also shows the point of the positively and negatively charged adsorbent on the internal surface. The zeta potential of the pCh-MWCNTs@Ag-TiO_2_ and pCh-MWCNTs@Ag-TiO_2_/S particles declines with increasing pH. By comparing the various observations in Figure 8, it can be concluded that at a pH below the point of zero charge, the removal efficiency of Hg^2+^ ions was low, and at a pH above the point of zero charge, the removal efficiency of Hg^2+^ was high. This is because at pH < pHpzc (point of zero charge) the adsorbent is positively charged, which causes the adsorbent to repel the adsorbate through electrostatic interactions, lowering the percentage removal of the Hg^2+^. On the other hand, at pH > pHpzc, the adsorbent is negatively charged and during the adsorption process, an electrostatic attraction ion exchange mechanism takes place between the adsorbent and adsorbate, increasing the percentage removal of the Hg^2+^ ion [23]. Furthermore, the increasing relative amounts of oxygen in the adsorbent particles shift the zeta potential gradually to the more negative range, which shows the larger amount of negative charges on the surface. The results revealed that pCh-MWCNTs@Ag-TiO_2_ has a higher absolute zeta potential value than pCh-MWCNTs@Ag-TiO_2_/S. This indicates that the surface of pCh-MWCNTs@Ag-TiO_2_ had more negative charges than pCh-MWCNTs@Ag-TiO_2_/S due to the presence of oxygen-containing groups such as carbonyl, carboxyl, and hydroxyl groups as shown in the FTIR spectra [36]. Therefore, these adsorbents exhibit higher dispensability and stability in water as well as a higher degree of functionality as functionalized composites [37].

#### 3.2.2. Effect of Solution pH

**Figure 9:**
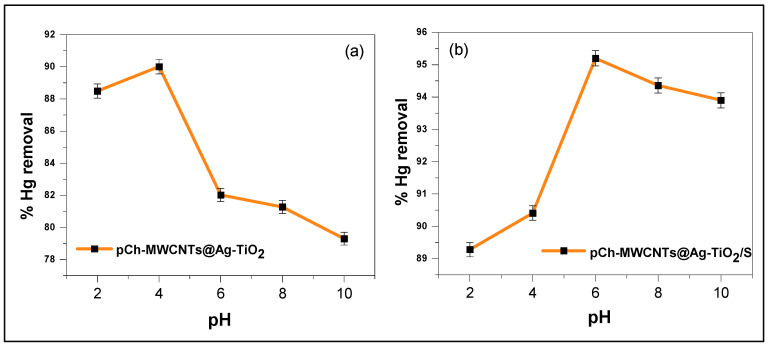
Effect of solution pH on the removal of Hg^2+^ by (**a**) pCh-MWCNTs@Ag-TiO_2_ and (**b**) pCh-MWCNTs@Ag-TiO_2_/S. Experimental conditions: Initial concentration 2 ppb, 24 h contact time, 0.0025 g amount of adsorbent, 25 °C temperature, and 30 mL solution volume.

The pH of the solution is very important in an adsorption process because it plays a role in the ionization of the functional groups present on the adsorbent surface and is responsible for trapping the target metal ion pollutants in water samples [38]. pH is also a critical factor in an adsorption system involving multifunctional charged macromolecules and metal ions because the pH can affect the dissociation of cation exchange groups, conformational changes in the molecular structure, the stability of metal complexes, and the speciation of metals [19,39,40]. Figure 9a,b illustrate how the pH of the solution was varied at pH 2, 4, 6, 8, and 10 to examine the adsorption of Hg (II) ions by pCh-MWCNTs@Ag-TiO_2_ and pCh-MWCNTs@Ag-TiO_2_/S. The adsorption capacities and behavior of these two materials, namely, pCh-MWCNTs@Ag-TiO_2_ and pCh-MWCNTs@Ag-TiO_2_/S, are completely different. In Figure 9a, a slight increase in removal rate between pH 2 to pH 4 is observed, while in Figure 9b, there is a rapid initial increase that reaches a maximum and levels off at around pH 6. Beyond this pH value, no further increase in the adsorption rate was observed. According to Haug (1961), groups below pH 4,5 (-COOH) are protonated; as a result, there is little electrostatic attraction between the positively charged cations and the adsorbent surface [41]. On the other hand, functional groups (-COOH, -OH, and -NH) on the surface of the adsorbent are deprotonated at high pH values (7 to 8.5), which enhances the adsorption of cations. As a result, the adsorbent’s surface had more negative charges, which enhanced the adsorption capacity and attraction forces. Wenliang et al. (2025) demonstrated that the material maintains strong stability across varying pH levels, a property linked to its consistently positive zeta potential, which promotes favorable electrostatic interactions with cations [42].

Furthermore, many studies have observed similar trends regarding the effect of pH on the adsorption of metal ions such as mercury, lead, and copper from aqueous solutions; this was attributed to the ionization of the surface functional groups depending on the charge of the metal ions to be adsorbed [43]. It is visible that the highest percent removal of mercury was achieved using pCh-MWCNTs@Ag-TiO_2_/S as an adsorbent. The optimum pH obtained for pCh-MWCNTs@Ag-TiO_2_ and pCh-MWCNTs@Ag-TiO_2_/S adsorbents were pH 4 and pH 6, respectively.

#### 3.2.3. Effect of Adsorbent Dosage

**Figure 10:**
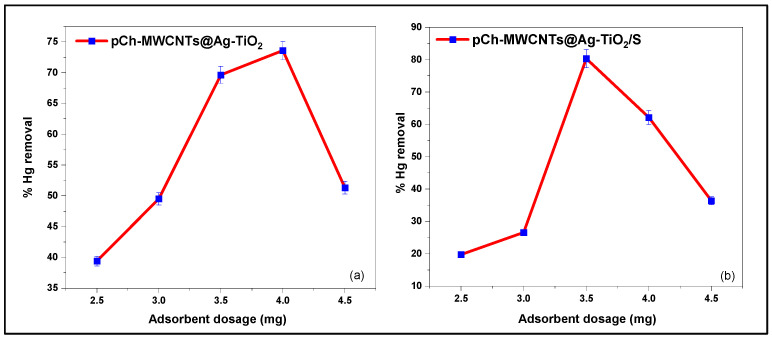
Effect of adsorbent dosage on % removal of Hg^2+^ concentration from aqueous solution. Experimental conditions: Initial concentration 2 ppb, 24 h contact time, 25 °C temperature, 30 mL solution volume, and pH [(**a**) pCh-MWCNTs@Ag-TiO_2_ = 4, (**b**) pCh-MWCNTs@Ag-TiO_2_/S = 6].

The effect of the adsorbent dose on the adsorption of the Hg^2+^ ions on the two different adsorbents was investigated (Figure 10). The plot shows the effect of adsorbent dosage on the percentage removal of Hg^2+^ by pCh-MWCNTs@Ag-TiO_2_ (Figure 10a) and pCh-MWCNTs@Ag-TiO_2_/S (Figure 10b) in an aqueous solution. The mass of the adsorbent varied from 2.5 to 4.5 mg while other parameters were kept constant. Figure 10 shows that the adsorption removal of mercury increases with an increase in the mass of the adsorbent from 2.5 to 4.5 mg until it reaches the optimum dosage and thereafter the removal percentage decreases. This showed that the adsorption sites remained unsaturated during the adsorption reaction because as the dosage of the adsorbent was increased, the adsorption sites for binding of the adsorbate also increased [44]. Conversely, other studies explain this behavior with a reduction in the active surface for adsorption [45]. Rao and co-workers, reported that this behavior is due to the formation of adsorbent aggregates at higher adsorbent dosages [46], thereby decreasing the effective surface area for adsorption [47]. For this study, the optimum mass of the adsorbent is 4.0 mg and 3.5 mg for pCh-MWCNTs@Ag-TiO_2_ (Figure 10a) and pCh-MWCNTs@Ag-TiO_2_/S (Figure 10b), respectively. The pCh-MWCNTs@Ag-TiO_2_/S shows a higher percent mercury removal of 80, 30%. This reveals that sulfur nanoparticles added to the polymer increase the adsorption strength of the polymer.

#### 3.2.4. Effect of Initial Concentration on Hg^2+^ Adsorption

**Figure 11:**
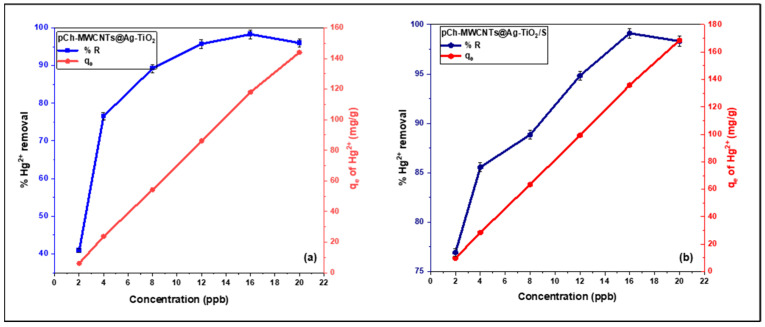
Effect of initial concentration on % removal of Hg^2+^ concentration from aqueous solution. Experimental conditions: 24 h contact time, adsorbent [(**a**) pCh-MWCNTs@Ag-TiO_2_ = 4 mg, (**b**) pCh-MWCNTs@Ag-TiO_2_/S = 3.5 mg], 25 °C temperature, 30 mL solution volume, and pH (pCh-MWCNTs@Ag-TiO_2_ = 4, pCh-MWCNTs@Ag-TiO_2_/S = 6).

The effect of initial concentration on the removal and adsorption capacity of Hg^2+^ by pCh-MWCNTs@Ag-TiO_2_ (Figure 11a) and pCh-MWCNTs@Ag-TiO_2_/S (Figure 11b) was investigated. The initial concentration was varied from 2 ppb to 20 ppb, and all other parameters were kept constant. The trend of the plots recorded an increase in adsorption with increasing cation solution concentrations. Moreover, it can be observed that the initial concentration increases rapidly with percentage removal until it reaches the highest removal or equilibrium values of 98.31% and 99.11% for pCh-MWCNTs@Ag-TiO_2_ and pCh-MWCNTs@Ag-TiO_2_/S, respectively. This can be attributed to the fact that the initial concentration of the cation solution represents an important driving force to overcome the mass transfer resistance of the cations between the aqueous and solid phases. Therefore, higher initial cation concentration results in higher adsorption. This shows that the initial concentration of Hg ion is directly proportional to the removal capacity of the adsorbent as shown in Figure 11a,b. Therefore, in this study, an initial metal concentration of 16 ppb is considered the optimal initial metal concentration for both polymers, pCh-MWCNTs@Ag-TiO_2_ and pCh-MWCNTs@Ag-TiO_2_/S. The respective adsorption capacities achieved at this initial metal concentration were 117.97 and 135.93 mg/g. In addition, among the two adsorbents, pCh-MWCNTs@Ag-TiO_2_/S has the higher adsorption capacity, as shown in Figure 11b.

Table 4 summarizes the maximum monolayer adsorption capacities of Hg^2+^ onto pCh-MWCNTs@Ag-TiO_2_ and pCh-MWCNTs@Ag-TiO_2_/S. The pCh-MWCNTs@Ag-TiO_2_ and pCh-MWCNTs@Ag-TiO_2_/S used in this study achieved a capacity of 117.97 and 135.93 mg/g, respectively, which is comparatively higher than many adsorbents reported in the literature. This relatively strong performance highlights the potential of pCh-MWCNTs@Ag-TiO_2_ and pCh-MWCNTs@Ag-TiO_2_/S as an efficient and competitive material for mercury removal applications.

#### 3.2.5. Effect of Contact Time

**Figure 12:**
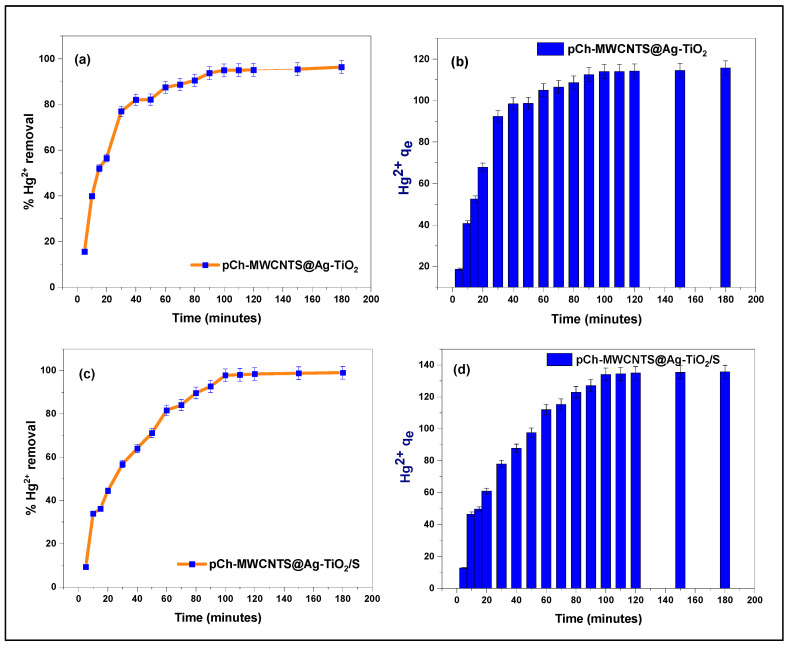
Effect of contact time on % removal (**a**,**c**) and capacity (**b**,**d**) of Hg^2+^ concentration from aqueous solution. Experimental conditions: Initial concentration (pCh-MWCNTs@Ag-TiO_2_ = 16 ppb, pCh-MWCNTs@Ag-TiO_2_/S = 16 ppb), pH (pCh-MWCNTs@Ag-TiO_2_ = 4, pCh-MWCNTs@Ag-TiO_2_/S = 6), and adsorbent dosage (pCh-MWCNTs@Ag-TiO_2_ = 4 mg, pCh-MWCNTs@Ag-TiO_2_/S = 3.5 mg).

The effect of contact time on the percentage removal of Hg^2+^ by pCh-MWCNTs@Ag-TiO_2_ and pCh-MWCNTs@Ag-TiO_2_/S is presented in Figure 12. The contact time was varied from 5 to 180 min while maintaining the other parameters constant. A trend of an increase in removal percentage with an increase in contact time was observed for both adsorbents. From 5 to 10 min, the Hg^2+^ ion percentage removal increased from 15 to 40% for pCh-MWCNTs@Ag-TiO_2_ (Figure 12a) and 9 to 33% for pCh-MWCNTs@Ag-TiO_2_/S (Figure 12c).

The adsorption dynamics of mercury (II) ions on these two adsorption materials were monitored at a temperature of 298 K and showed rapid mercury (II) uptake (Figure 12). Each adsorbent reached equilibrium at different times. For instance, pCh-MWCNTs@Ag-TiO_2_ (Figure 12a), reached equilibrium at 90 min, and pCh-MWCNTs@Ag-TiO_2_/S (Figure 12c) at 100 min. The notable observation here is that pCh-MWCNTs@Ag-TiO_2_ reaches equilibrium after 90 min, which was faster than that of pCh-MWCNTs@Ag-TiO_2_/S. However, it can also be observed that the percentage removal of mercury ions by pCh-MWCNTs@Ag-TiO_2_/S is higher than that of pCh-MWCNTs@Ag-TiO_2_. From 90 min to 180 min (pCh-MWCNTs@Ag-TiO_2_), and 100 min to 180 min (pCh-MWCNTs@Ag-TiO_2_/S), both adsorption materials were saturated and could not allow further adsorption of the mercury ions. The plot showed that a further increase in contact time did not result in a significant increase in adsorption. This behavior is because, at the beginning of the process, a large number of functional groups of amines, amides, and hydroxyl, carboxyl, and ketone groups of the adsorbents are available in the form of active adsorption sites [53]. However, with time, the number of occupied active sites decreases, so that the adsorption rate reaches an equilibrium stage. A similar phenomenon has been observed by many other authors [54,55]. Hence, the optimum contact times in this study were 90 and 100 min for pCh-MWCNTs@Ag-TiO_2_ and pCh-MWCNTs@Ag-TiO_2_/S, respectively.

#### 3.2.6. Effect of Temperature

**Figure 13:**
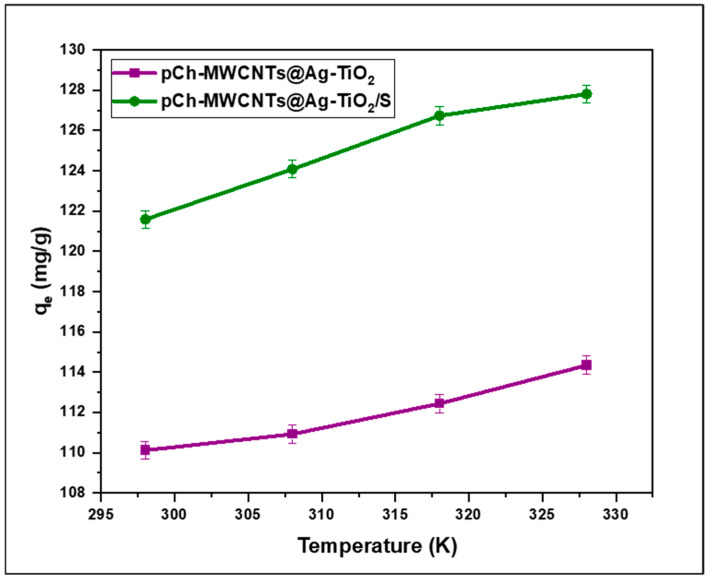
Effect of temperature on % removal of Hg^2+^ concentration from aqueous solution. Experimental conditions: Initial concentration (pCh-MWCNTs@Ag-TiO_2_ = 16 ppb, pCh-MWCNTs@Ag-TiO_2_/S = 16 ppb), and pH (pCh-MWCNTs@Ag-TiO_2_ = 4, pCh-MWCNTs@Ag-TiO_2_/S = 6, pCh-MWCNTs@Ag-TiO_2_ = 4 mg, pCh-MWCNTs@Ag-TiO_2_/S = 3.5 mg).

The influence temperature on the removal of Hg (II) by pCh-MWCNTs@Ag-TiO_2_ and pCh-MWCNTs@Ag-TiO_2_/S was studied at 298 K, 308 K, 318 K, and 328 K at an optimum initial concentration of each adsorbent, and the results are shown in Figure 13. It has been shown that temperature is one of the most essential parameters affecting the adsorption capacity of an adsorbent [5,9,11,28]. From this behavior, it is observed that as the temperature increases, the mercury adsorption capacity also increases. The linear adsorption trend in pCh-MWCNTs@Ag-TiO_2_ and pCh-MWCNTs@Ag-TiO_2_/S adsorbents (Figure 13), shows a significant increase in adsorption capacity from 110.12 mg/g to 114.63 mg/g and 121.58 mg/g to 127.83 mg/g, respectively, as temperature increased from 298 K to 328 K. This indicates that the reactions are endothermic. Furthermore, it is known that an increase in temperature due to the decrease in viscosity of the solution increases the rate of diffusion of adsorbate molecules across the outer boundary layer and into the internal pores of the adsorbent particle. A change in temperature changes the equilibrium capacity of the adsorbent for a particular adsorbate [56]. This may be because the mobility of Hg ions increases with increasing temperature. An increasing number of molecules can also receive sufficient energy to interact with active sites on the surface. Furthermore, the increasing temperature can lead to a swelling effect within the internal structure of the adsorbents, allowing large Hg ions to further penetrate [40,57].

### 3.3. Adsorption Isotherm Modelling

The adsorption isotherms are very important in adsorption studies because they indicate how the adsorption molecules distribute between the liquid phase and the solid phase when the adsorption process reaches equilibrium. Analyzing the isotherm data by fitting them to different isotherm models is an important step in finding the appropriate model that can be used for design purposes [58]. In this study, two adsorption isotherm models (Langmuir and Freundlich) were used to describe the experimental data in more detail such as equilibrium or maximum capacity, percentage removal and interaction behaviors (adsorption mechanism) of mercury on pCh-MWCNTs@Ag-TiO_2_ and pCh-MWCNTs@Ag-TiO_2_/S.

#### 3.3.1. Langmuir and Freundlich Isotherms

**Figure 14:**
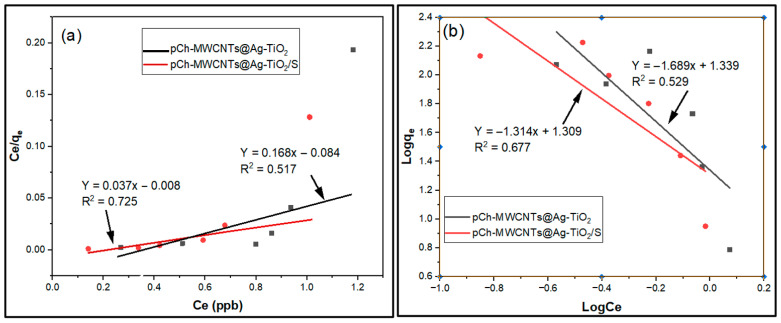
Langmuir (**a**) and Freundlich (**b**) adsorption isotherm plot for adsorption of mercury ions onto pCh-MWCNTs@Ag-TiO_2_ and pCh-MWCNTs@Ag-TiO_2_/S.

The Langmuir and Freundlich isotherm graphs for the adsorption of Hg ions on both pCh-MWCNTs@Ag-TiO_2_ and pCh-MWCNTs@Ag-TiO_2_/S were determined using the obtained data points as shown in Figure 14a,b. The correlation coefficients (*R*^2^) obtained for the Langmuir isotherm model were 0.517 and 0.725 for pCh-MWCNTs@Ag-TiO_2_ and pCh-MWCNTs@Ag-TiO_2_/S, respectively. The adsorption had a poor fit for both mechanism process. Although, pCh-MWCNTs@Ag-TiO_2_/S had a higher *R*^2^ value compare to pCh-MWCNTs@Ag-TiO_2_, the adsorption process does not follow Langmuir isotherm. Freundlich isotherm models were 0.529 and 0.677 for pCh-MWCNTs@Ag-TiO_2_ and pCh-MWCNTs@Ag-TiO_2_/S, respectively, as shown in Figure 14b. The Freundlich adsorption also had a poor fit for both mechanisms, an indication that the process does not follow the Freundlich isotherm model.

As shown in Table 5, the correlation coefficient (*R*^2^) and residual standard error (*RSE*) were used to derive which isotherm model best fit the results.

The values of *k_f_* and n were calculated from the slope and intercept of the graph. Therefore, the values of *k_f_* were 0.424 (pCh-MWCNTs@Ag-TiO_2_) and 0.268 (pCh-MWCNTs@Ag-TiO_2_/S), and the values of n were −1.69 and −1.31 for the adsorption of mercury ions on pCh-MWCNTs@Ag-TiO_2_ and pCh-MWCNTs@Ag-TiO_2_/S, respectively. This indicates that the adsorption process was not favorable under the studied conditions [59].

The maximum Langmuir adsorption capacity *(q_max_*), shown in Table 4, was 5.96 mg/g and 26.74 mg/g for pCh-MWCNTs@Ag-TiO_2_ and pCh-MWCNTs@Ag-TiO_2_/S, respectively. The difference in the maximum Langmuir adsorption capacity of the monolayer between pCh-MWCNTs@Ag-TiO_2_ and pCh-MWCNTs@Ag-TiO_2_/S shows that the activation process plays an essential role in improving the adsorption properties of the adsorbent materials which lead to an increased adsorption capacity.

In this regard, the high *R*^2^ (0.725) and low *RSE* (0.09822) in Langmuir were consistent with the visual observation that the process can be attempted to be described by a Langmuir adsorption isotherm, even though failed. Additionally, the Langmuir separating factor (*R_L_*) was not between 0 < *R_L_* < 1 (the Langmuir separation factor was calculated to be −0.03 and −0.01 for pCh-MWCNTs@Ag-TiO_2_ and pCh-MWCNTs@Ag-TiO_2_/S, respectively), suggesting that the model or adsorption process is unfavorable and was irreversible [19].

Table 5 shows the isotherm constants obtained for the two adsorption models. The correlation coefficient (*R*^2^) and residual standard error (*RSE*) were used to derive which isotherm model best fit the results. From the table, the Langmuir isotherm model shows the best agreement with the experimental data for pCh-MWCNTs@Ag-TiO_2_/S and achieved correlation coefficients of 0.725 for the adsorption of mercury ions. This implies that the adsorption of mercury ions on pCh-MWCNTs@Ag-TiO_2_/S is limited to the mono-layer covering of the surface of the adsorbent with the adsorbate, and that the interaction between the adsorbents and the mercury ions allows the formation of mono-layers. However, neither adsorption process follows Langmuir or Freundlich assumptions.

#### 3.3.2. Adsorption Kinetics

Adsorption kinetics is crucial in determining the residence time required to complete the adsorption process because it provides essential information about the reaction pathways and solute uptake rate. The pseudo-first-order (PFO), pseudo-second-order (PSO), Elovich, and intraparticle diffusion (IPD) models were used to determine the kinetic studies of Hg (II) removal by pCh-MWCNTs@Ag-TiO_2_ and pCh-MWCNTs@Ag-TiO_2_/S.

##### Pseudo-First Order and Second Order

**Figure 15:**
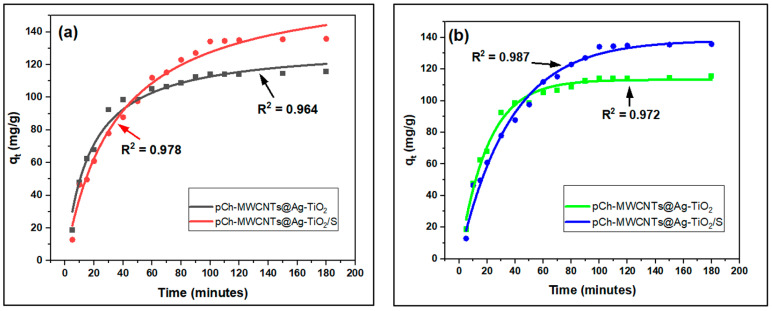
(**a**) PFO and (**b**) PSO kinetic model plot for adsorption of mercury ions on pCh-MWCNTs@Ag-TiO_2_ and pCh-MWCNTs@Ag-TiO_2_/S.

The pseudo-first-order (PFO) and pseudo-second-order (PSO) models were used to investigate the kinetics of the adsorption processes. When metal ions diffuse onto the adsorbent boundary layer, an adsorption mechanism known as pseudo-first order is created [60]. PFO (Figure 15a) and PSO (Figure 15b) are empirical models, whereby few physical meanings are expressed [61]. PFO kinetic model is considered one of the earliest kinetic models to describe the adsorption of solids in solid-liquid systems [62], and is expressed by Equation (6). On the other hand, PSO predicts that the adsorption process occurs at the final stage of the reaction where equilibrium is reached. It also suggests that the rate-determining step is controlled by chemical adsorption, which involves violent forces consisting of electron sharing and exchange between the adsorbent and the adsorbate [62]. The kinetic PSO model is described by Equation (7). According to Figure 15a, both processes had a good fit, with *R*^2^ = 0.964 and *R*^2^ = 0.978 for pCh-MWCNTs@Ag-TiO_2_ and pCh-MWCNTs@Ag-TiO_2_/S, respectively. Figure 15b reveals that the process had an excellent fit, with *R*^2^ = 0.987 for pCh-MWCNTs@Ag-TiO_2_/S, and *R*^2^ = 0.972 for pCh-MWCNTs@Ag-TiO_2_. Due to the fact that Figure 15b shows the best overall fit, this confirmed that the process is best described by PSO, meaning chemisorption is the dominant mechanism with adsorption rate proportional to the square of unoccupied sites.

In addition, PSO also predicts that the adsorbent consists of relatively numerous active sites. The model also follows the Langmuir adsorption isotherm model regarding the monolayer coverage of the adsorbate on the surface of the adsorbent [63].

##### Elovich Model

**Figure 16:**
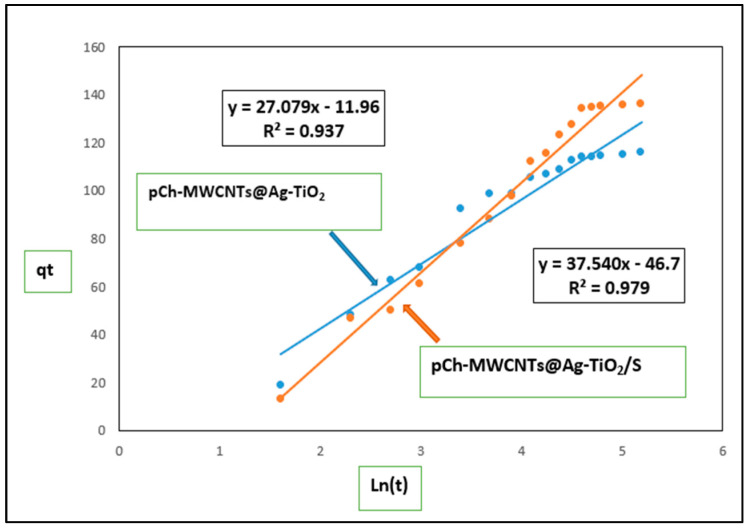
Elovich model for adsorption of mercury ions onto pCh-MWCNTs@Ag-TiO_2_ and pCh-MWCNTs@Ag-TiO_2_/S.

The Elovich model is an empirical approach that is commonly employed to describe chemisorption processes [29]. According to this model, the activation energy required for adsorption tends to rise as the adsorption progresses, suggesting that the process takes place on a heterogeneous surface [64]. The higher determination coefficient (*R*^2^), compared with those of the PFO and PSO models, further indicates that the mechanism is governed by chemisorption. Within this model, the constant α represents the initial adsorption rate, while *β* reflects the extent of surface coverage, which is also related to the desorption rate [65]. Figure 16 shows that the Elovich model still works well since it also provides the best fit, especially for pCh-MWCNTs@Ag-TiO_2_/S. Since PSO and Elovich model fit well, this confirms that the adsorption is mainly chemisorption, occurring on a heterogeneous surface.

According to Table 6, *α* (initial adsorption rate) for pCh-MWCNTs@Ag-TiO_2_ is higher compared to pCh-MWCNTs@Ag-TiO_2_/S, indicating that the adsorption process starts at a rapid initial uptake. However, *β* = 0.037 and 0.027 for pCh-MWCNTs@Ag-TiO_2_ and pCh-MWCNTs@Ag-TiO_2_/S, respectively. This shows how quickly adsorption process slows as the surface becomes occupied. Larger *β* implies stronger surface heterogeneity and slower desorption [64]. The model suggests adsorption is slower at later stages because surface sites become less available.

##### Intraparticle Diffusion (IPD)

The intraparticle diffusion (IPD) kinetic model estimates the type of process, which could be pores or surface adsorption. The parameter *C* (mg/g) values measure the degree of thickness of the accumulated cations adsorbate on the adsorbents surface. In addition, the adsorption process occurs through various stages that include the transport of the adsorbate from the solution to the surface of the adsorbent [62,65].

**Figure 17:**
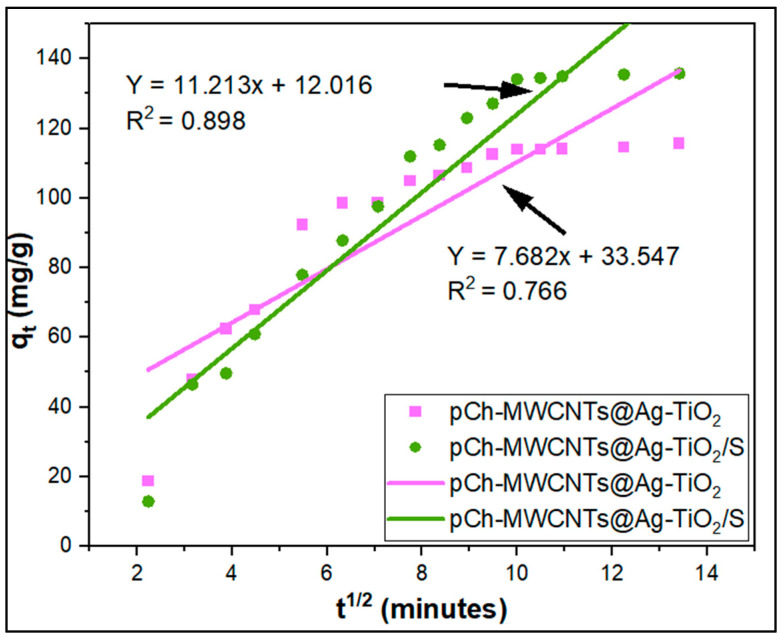
Intraparticle diffusion graph of pCh-MWCNTs@Ag-TiO_2_ and pCh-MWCNTs@Ag-TiO_2_/S.

Furthermore, the kinetic model of intraparticle diffusion proposed by Weber and Morris has been widely used to study the rate-limiting step in the adsorption of mercury, as shown in Figure 17. A plot of *q_t_* vs. *t*^1/2^ must be a straight line passing through the origin, where *k_d_* is the slope of the plot so that diffusion within the particles is the only rate-limiting step. However, it is not always the case that a plot of *q_t_* vs. *t*^1/2^ passes through the origin, suggesting that intraparticle diffusion is not the only rate-determining step, but that adsorption kinetics are controlled by film diffusion in the initial stage of the subsequent reaction and can be achieved by intraparticle diffusion later during the reaction. A plot of *q_t_* vs *t*^1/2^ in Figure 17 was used to determine the velocity control step. The plot showed multiple linearity, showing that intraparticle diffusion is not the only rate-controlling step in the adsorption process. This means that the reaction rate was controlled by both film diffusion through a boundary layer and intraparticle diffusion [65].

The correlation coefficients obtained for the intraparticle diffusion kinetics model were 0.898 and 0.766 for the adsorption of mercury on pCh-MWCNTs@Ag-TiO_2_ and pCh-MWCNTs@Ag-TiO_2_/S, which were lower than both the pseudo-first-order kinetic model and the pseudo-second-order kinetic model. Since the plot of *q_t_* vs. *t*^1/2^ does not pass through the origin, it means that intraparticle diffusion is not the only rate-determining step but may be accompanied by film diffusion [66].

#### 3.3.3. Thermodynamics Studies

**Figure 18:**
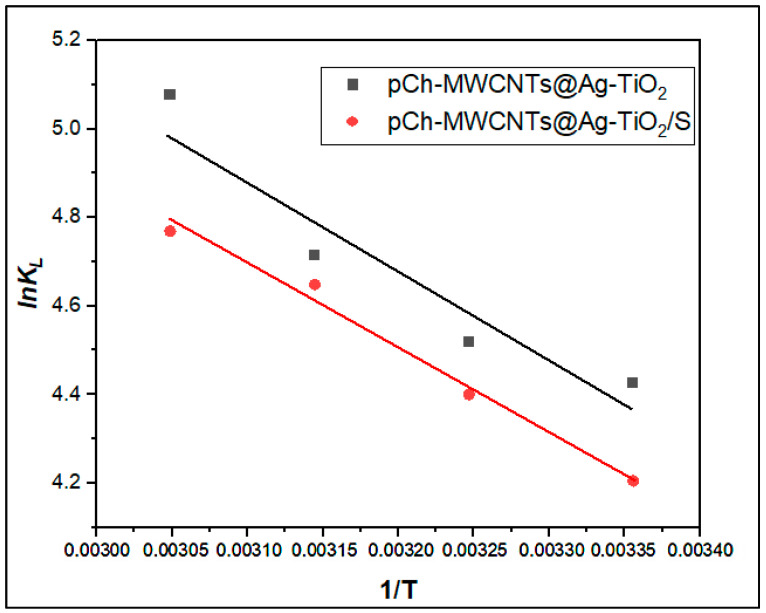
Plot of *lnK_L_* vs. *1*/*T* for estimation of adsorption thermodynamic parameters for adsorption of mercury ions onto pCh-MWCNTs@Ag-TiO_2_ and pCh-MWCNTs@Ag-TiO_2_/S.

The thermodynamic parameters such as enthalpy (Δ*H*°), Gibbs free energy (Δ*G*°), and entropy (Δ*S*°) were assessed to determine the spontaneity and feasibility of Hg (II) ions sorption onto pCh-MWCNTs@Ag-TiO_2_ and pCh-MWCNTs@Ag-TiO_2_/S. The plot of *lnK_L_* vs. *1*/*T* (Figure 18) was used to obtain the values of the thermodynamic parameters Δ*H*° and Δ*S*°.

The values of Δ*H*° and Δ*S*° were calculated for the adsorption of mercury ions on both adsorbents as shown in Table 7. The parameters were evaluated at different temperatures (298, 308, 318, and 328 K). The Gibbs free energy (Δ*G*°) had negative values for all materials, indicating that all adsorption processes were spontaneous and feasible. Furthermore, a study by Zhao et al. (2016) presented a trend in which Δ*G*° decreased with increasing temperatures and concluded that Hg uptake was temperature dependent [34]. To support the study by Zhao et al. (2016) [34], the enthalpy (Δ*H*°) provided positive values to confirm that Hg adsorption is an endothermic reaction. Furthermore, the entropy Δ*S*° had positive values, indicating increased randomness and degree of freedom for cations in aqueous solution during the adsorption process. Therefore, the adsorption of Hg^2+^ ions on pCh-MWCNTs@Ag-TiO_2_ and pCh-MWCNTs@Ag-TiO_2_/S are values of thermodynamic adsorption parameters Δ*H*°, Δ*S*°, and Δ*G*° at different temperatures are listed in Table 7.

## 4. Conclusions

In this research, pCh-MWCNTs@Ag-TiO_2_ and pCh-MWCNTs@Ag-TiO_2_/S adsorbents were successfully synthesized, as confirmed by comprehensive characterization using SEM/EDS, FTIR, TGA, BET analysis, and Zeta potential measurements. FTIR spectra confirmed the presence of functional groups such as hydroxyl (-OH), carboxyl (-COOH), and sulfur functionalities in the structure of pCh-MWCNTs@Ag-TiO_2_/S. SEM/EDS imaging revealed that the nanocomposites, particularly pCh-MWCNTs@Ag-TiO_2_/S and pCh@S, exhibited spherical and agglomerated structures, suggesting the formation of a distinct nanoparticle phase on the surface of the composites. The high adsorption capacity is attributed to the strong, specific chemisorption of mercuric ions onto sulfur-containing functional groups on the adsorbent’s surface. This Hg-S binding, driven by the soft–soft acid–base interaction, is theorized to be the primary mechanism, resulting in the formation of stable complexes (e.g., Hg-SR_2_).

The Hg^2+^ adsorption performance of both materials was systematically evaluated. Key factors influencing adsorption efficiency including pH, dosage of the adsorbent, contact time, initial mercury concentration, and temperature were optimized to determine the most effective conditions. The adsorption behavior was further analyzed using isotherm and kinetic models to understand the underlying mechanism. Among the two materials, pCh-MWCNTs@Ag-TiO_2_/S demonstrated superior performance, achieving a maximum adsorption capacity of 26.74 mg/g based on the Langmuir model. However, the Langmuir separation factor (*R_L_*) and Freundlich constant (*n_f_*) fell outside their expected favorable ranges, indicating that the adsorption process did not align well with the assumptions of these models.

Kinetic modeling showed that the PSO model best described the adsorption process, implying that chemisorption is the rate-controlling step. In addition, Elovich model fitted well, confirming that the adsorption is mainly chemisorption, occurring on a heterogeneous surface. Thermodynamic analysis was conducted to assess the spontaneity, feasibility, and nature of the mercury adsorption. The results indicated that the adsorption process is endothermic and occurs spontaneously, with increased disorder at the solid–liquid interface during interaction with Hg^2+^ ions.

## 5. Recommendations/Future Work

For future research, it is recommended to investigate the applicability of these biopolymer-based adsorbents for capturing other hazardous contaminants and to compare their effectiveness across a broader range of pollutants. A key limitation of this study is the investigation of Hg^2+^ removal only in single-component systems, which does not account for the complex matrix of real wastewater containing competing ions and organic matter. Therefore, to fully assess the practical potential of biopolymer-based adsorbents, future work must include competitive adsorption experiments with common ions (e.g., Pb^2+^, Cd^2+^, Cu^2+^, Ca^2+^, Cl^−^, SO_4_^2−^), tests using real industrial wastewater, and an evaluation of the adsorbent’s selectivity, regeneration, and reusability in these complex environments.

## Figures and Tables

**Table 1:** List of chemicals used and their grades.

Chemicals	Purity	Supplier
Raw chitosan	≥75%	Sigma Aldrich, Johannesburg, South Africa
Pristine multiwalled carbon nanotubes	90%	Sigma Aldrich, Johannesburg, South Africa
Phosphoric acid (H_3_PO_4_)	85%	Sigma Aldrich, Johannesburg, South Africa
1-butanol	99.8%	Sigma Aldrich, Johannesburg, South Africa
Triethyl phosphate (Et_3_PO_4_)	99.8%	Sigma Aldrich, Johannesburg, South Africa
Phosphorous pentoxide (P_2_O_5_)	99%	Sigma Aldrich, Johannesburg, South Africa
Silver nitrate	99.8	Rochelle Chemicals, Johannesburg, South Africa
Acetone	99.5%	Sigma Aldrich, Johannesburg, South Africa
Titanium isopropoxide (TTIP)	97%	Sigma Aldrich, Johannesburg, South Africa
N,N-Dimethylformamide (DMF)	99.9%	Sigma Aldrich, Johannesburg, South Africa
Hexamethylene diisocyanate (HMDI)	98.0%	Sigma Aldrich, Johannesburg, South Africa
Sulfuric acid (H_2_SO_4_)	98.08%	Rochelle chemicals, Johannesburg, South Africa
Nitric acid (HNO_3_)	55%	Rochelle chemicals, Johannesburg, South Africa
Mercury chloride (HgCl_2_)	99.5%	Scharlau, Barcelona, Spain
Hydrochloric acid	33%	Glass world, Johannesburg, South Africa

**Table 2:** Mass percentage of detected elements of pCh-MWCNTs@Ag-TiO_2_/S.

pCh-MWCNTs@Ag-TiO_2_/S		
**Elements**	**Weight (%)**	**Atomic (%)**
C	74.19	82.59
O	17.48	14.61
Si	0.88	0.42
P	1.06	0.46
S	1.49	0.62
Ti	4.46	1.25
Ag	0.42	0.05
Total	100	-
**pCh-S**		
C	21.75	42.59
Si	0.13	0.11
S	78.12	57.30
Total	100	-
**S**		
S	100	100
Total	100	-

**Table 3:** Results summary of BET surface area analysis.

Adsorbents	BET Surface Area (m^2^/g)	Pore Volume (cm^3^/g)	Pore Size (nm)
pCh-MWCNTs@Ag-TiO_2_	0.578	0.205	1811.71
pCh-MWCNTs@Ag-TiO_2_/S	3.506	0.262	234.37

**Table 4:** Comparison of maximum adsorption capacity of Hg^2+^ on to various adsorbents.

Adsorbents	Adsorption Capacity (mg/g)	References
pCh-MWCNTs@Ag-TiO_2_	117.97	This work
pCh-MWCNTs@Ag-TiO_2_/S	135.93	This work
Sulfur-doped reduced graphene oxide@chitosan composite	78.9	[48]
Thiol-Functionalized Graphene Oxide	98	[49]
Novel Activated Carbon-Based Composite	289	[50]
Novel Composite of Polyacrylate-Modified Carbon	76.3	[51]
Biochar fabricated with steel slag	283.24	[52]

**Table 5:** Langmuir and Freundlich adsorption isotherm parameters.

Isotherms	Parameters	pCh-MWCNTs@Ag-TiO_2_	pCh-MWCNTs@Ag-TiO_2_/S
Langmuir	*q_max_*	5.957	26.738
$q_{t}=\frac{q_{m}bC_{e}}{\left(1+bC_{e}\right)}$	*K_L_*	−2.004	−4.589
	*R_L_*	−0.032	−0.014
	*R* ^2^	0.517	0.725
	*RSE*	0.013	0.09822
Freundlich	*n_f_*	−1.689	−1.314
$q_{e}=k_{f}{C_{e}}^{n}$	*k_f_*	0.825	0.981
	*R* ^2^	0.529	0.677
	*RSE*	0.519	0.397

**Table 6:** Adsorption kinetics constant for adsorption of mercury ions on pCh-MWCNTs@Ag-TiO_2_ and pCh-MWCNTs@Ag-TiO_2_/S.

Models	Parameters	pCh-MWCNTs@Ag-TiO_2_	pCh-MWCNTs@Ag-TiO_2_/S
Pseudo-first order (PFO)	*q_e_*	113.31	138.31
	*K*_1_	0.0493	0.0277
	*R* ^2^	0.964	0.978
Pseudo-second order (PSO)	*q_e_*	131.74	172.18
	*q_e_* ^2^	17,355.34	29,645.95
	*K*_2_	0.0449	0.0166
	*R* ^2^	0.972	0.987
Elovich	*β*	0.037	0.027
	*α*	17.414	10.820
	*R* ^2^	0.937	0.979
Intraparticle diffusion (IPD)	*K_d_*	7.682	11.212
	*C*	33.547	12.016
	*R* ^2^	0.766	0.898

**Table 7:** Thermodynamic parameters for Hg adsorption.

Adsorbents	Temp (K)	$K_{L}=\frac{q_{e}}{C_{e}}$	∆G° (KJ/mol)	∆H° (KJ/mol)	∆S° (J/(k mol))	$R^{2}$
pCh-MWCNTs@Ag-TiO_2_	298	83.7	−10.9	17.3	94.3	0.87
	308	91.7	−11.6			
	318	111.6	−12.5			
	328	160.3	−13.8			
pCh-MWCNTs@Ag-TiO_2_/S	298	67.0	−10.4	15.8	88.0	0.98
	308	81.4	−11.3			
	318	104.4	−12.3			
	328	117.8	−13.0			

## Data Availability

The data that support the findings of this study are available from the corresponding author, M.J.K., upon reasonable request.
